# Clinic of Jefferson Medical College

**Published:** 1852-07

**Authors:** J. Aitken Meigs


### CLINICAL REPORTS.

*Clinic of Jefferson Medical College. Service of* PROFESSOR DUNGLISON. *February* 21*st*, 1852.

(Reported by J. AITKEN MEIGS, M. D.)

I am, to-day, gentlemen, enabled, through the kindness of Mr. Bates, of this city, who is present, to bring before you three cases illustrative of the nature and treatment of that form of defective or imperfect speech, known as "stammering." It is a subject not often, perhaps, examined in a clinic, yet it is unquestionably a morbid condition, and no better opportunity than the present could offer itself to explain to you its phenomena, and to investigate their causes. Mr. Bates, who has the three persons now before you under his care, with that candor and liberality which ought to characterize every one connected directly or indirectly with our noble avocation, had expressed, when I proposed the matter to him, on his calling to explain his views on impediments of speech to me, his entire willingness to permit me not only to exhibit some of his cases to you, but also to be the expounder of the methods he adopts for its rectification; and, after having done so, I shall endeavor to deduce for you the inferences at which I have arrived in regard to the *modus operandi* of his means and appliances, and to the great principles of management that flow therefrom.

To-day, such an examination and inquiry are especially appropriate, as I have been engaged, during the past week, in another place, in expounding to you the physiology of phonation, and the modes in which the different vocal sounds are elicited in the glottis, and modified in the vocal tube. The vowels, as you well know, flow without obstacle, and consequently demand no consideration. They are simple modifications of the voice formed in the larynx, and are uninterrupted by the organs—as the tongue, lips, &c.—in their passage through the vocal tube; while the consonants require different, and, at times, complex and delicate movements of the tube; and, as their name imports, have to be sounded with the vowels.

Stammering is a temporary inability to enunciate, freely and distinctly, certain letters at the commencement of one or more of the syllables of a word. There is a broken or interrupted emission of the voice in the act of articulation, and a consequent disconnection of the sounds; and you will understand, that the consonants must afford great obstacles to the stammerer, as they do, also, to children learning to talk; inasmuch as they are necessarily more difficult of enunciation than the vowels, in consequence of being dependent upon an ever-varying disposition and arrangement of the parts composing the vocal tube. Especially is this the case with that class of consonants known as explosives—as *b*, *d*, *t*, *g*, *k*, &c. These letters have of themselves no sound, or are mutes. They do not admit of a continuous pronunciation like the *h*, *m*, *n*, *f*, *s*, *r*, *l*, but require to be associated with a vowel sound, before they can be enunciated.

Much difference of sentiment, you will find, has existed in regard to the essential cause of stammering; and views have occasionally been entertained, which are certainly far from tenable. By some of the best physiologists, all the varieties have been referred to a spasmodic closure of the glottis producing a sudden arrestation of the issuing column of air. That this is not always the cause of the affection, however, is shown, as we shall see, by the cases before us. The great fault lies in the spasmodic action of certain of the muscles concerned in the production of the voice, and in articulation. Often, as in Chorea or St. Vitus's dance, the slightest agitation serves to aggravate, in the most painful degree, the abnormal action. Indeed, the affection may not improperly be—as it has been—called, "Chorea" or "St. Vitus's dance" of the voice. The stammerer, on attempting to enunciate a word or syllable, experiences difficulty or resistance at the commencement, and having but an imperfect control over the voluntary muscles of the vocal apparatus, he at once loses all confidence in his ability to produce the sound required, and there consequently results an irregular or spasmodic action of those muscles, which, for a longer or shorter period and determined by the degree of spasm, effectually prevents enunciation. In the case of the explosive consonants, the total interruption of the breath, and the badly regulated and insufficient volition, give occasion to the most painful spasmodic efforts on the part of the muscles more immediately concerned in articulation. This may be even extended to the whole body, which is thrown into a most distressing state of agitation to overcome the obstacle. At length the spasm ceases with the accomplishment of the act of expiration. It will now, therefore, be understood, why the complete interruption to expiration in the enunciation of the explosive consonants should be the most common phenomenon observed in stammerers. In the case, however, of the continuous consonants, an additional phenomenon occurs, in the sound being prolonged by spastic action for a much longer time than necessary.

Mr. Bates, who is an ingenious and liberal mechanic, has been studying, for some time, the nature and treatment of these distressing impediments to speech, and, as I remarked, has been kind enough to bring here several of the persons now under his care, that you may see me examine them, and hear me explain the mechanical contrivances which he employs to obviate them. He was himself, for a long time, a most intense sufferer, and, in consequence, had his attention earnestly and assiduously directed to the discovery of some means of relief. He has overcome the difficulty in himself, and has happily succeeded in enabling others to do the same. In the three cases now before you, and which are at present under his guidance, the spasm manifestly affects different muscles ; and hence, although in each person the same amount of difficulty is perhaps experienced in enunciation, the difficulty may concern different sets of letters. Thus the resistance may more prominently affect the labials, dento-labials, linguo-dentals, linguo-palatals, or gutturals ; and hence the value of the physiological knowledge which teaches us the intimate mode of their production.

[The patients were now brought, *seriatim*, before the class, and made to read words and syllables commencing with different consonants, especially with those of the explosive class.]

In the *first case*, (R—— G——, æt. 26,) the utterance of the explosive letters is arrested, and accompanied by a singular and sudden spasmodic protrusion of the lower lip. In the attempt to articulate such words as *Boston*, *bunch*, *boat*, *pill*, *pant*, *Pope*, &c. an arrestation of the sounding breath occurs, accompanied by such protrusion, and the patient is thus rendered incapable of completing or perfecting the sound.

In the *second case* (R—— S——, æt. 34) the voice is arrested, and there is a sudden and energetic contraction of the lips. The voice cannot escape from the mouth, and the difficulty here is with those words which contain the dentals, as *Thomas*, *Doctor*, *stone*, *&c.*

In the *third case* (D—— D——, æt. 25) there is spasm of the muscles that close the glottis, so that on attempting to pronounce the gutturals in such words as *grey*, *goose*, *great*, *king*, *court*, &c., the glottis is quickly and spasmodically closed, and the current of air prevented from issuing, except by jerks.

The great object to be accomplished in the treatment of these cases, is to overcome the proximate cause—the neurosis, or irregularity of innervation, indicated by the spastic condition of the muscular apparatus brought into play in the process of articulation. To effect this, it appeared to Mr. Bates, and it was confirmed by experiments instituted on himself whilst suffering under the infirmity, that if a plan could be imagined to prevent the total interruption of expiration, which occurs in these cases, the patient would feel confidence in his being able to elicit the particular sound, and in this manner the spasmodic efforts might be prevented. He accordingly invented several well devised instruments and arrangements, adapted to the different varieties of stammering; either by preventing the spasmodic action of the muscles concerned, or by restraining, by appropriate pressure, the irregular contractions of the muscles. For example, when the lower lip and chin, in the *first case*, were confined by means of a simple broad bandage, like the one I show you, and pressure was thus exerted upon the spasmodically contracted musculus orbicularis oris of the lower lip, so as to prevent the protrusion of the lips, the letters, which were such stumbling blocks before, could be distinctly enunciated, and with a daily decreasing amount of hesitation.

For the *second case* Mr. Bates has contrived, as you here see, a small plate, fitting closely to the palate, and affording attachment to a light narrow tube, the posterior end of which opens into the mouth, looking towards the fauces, whilst the anterior projects between the lips. By this contrivance the current of air is made to be in part continuous, and the patient finds, to his surprise and delight, that he can produce the sound without any limitation other than his will.

The subject of the *third case* has been materially benefited, and is, indeed, in a fair way to be entirely cured of his unfortunate habit, by means of a neckerchief or cravat, in which is a little spring, pressing—as you observe—directly upon the projection of the thyroid cartilage, in such a manner as to relax the rima glottidis, by approximating the thyroid to the arytenoid cartilages; thus permitting the exit of air and preventing the spasmodic action of the muscles that close the glottis. The spring is so regulated, that the amount of pressure upon the thyroid cartilage can be increased or diminished, as occasion may require.

[The effects of these different forms of apparatus were exhibited on the stammerers before the class; and the action of each was clearly manifested.]

By such contrivances, which are simple, and adapted to the accomplishment of the object in view, Mr. Bates succeeds in effecting a great desideratum,—the *restoration of self-confidence*,—the want of which is a main obstacle to improvement in all such cases; for as soon as the patient becomes thoroughly and practically convinced that there is no difference between his vocal organs and those of his friends, whom he hears speak without difficulty or hesitation, he becomes inspired with confidence in himself, and his exertions are thenceforth the commencement of his restoration. Let it be, however, distinctly understood—and no one is more satisfied of this truth than Mr. Bates himself—that no apparatus of this, or any kind, can, of itself, accomplish a cure. Nothing but a thorough system of practice and discipline, persevered in for a considerable period, can effect the work of restoration. The instrument merely infuses confidence. Withdraw it, and the case is left in the same condition as before. I can have no doubt, however, that with such a system, the stammerer may be amazingly benefited by the course pursued by Mr. Bates; but he must be thoroughly impressed with the fact, that time is one of the elements that must enter into his calculations, and that no plan can succeed without it.

